# Morphology and immunolocalization of intertubular steroidogenic cell in mesonephros of *Podocnemis expansa* during gonadal differentiation

**DOI:** 10.1590/1984-3143-AR2022-0011

**Published:** 2022-09-05

**Authors:** Luã Barbalho de Macêdo, Marcela dos Santos Magalhães, Lucas Castanhola Dias, Khelven Klay de Azevedo Lemos, Ryshely Sonaly de Moura Borges, Márcia Viviane Alves Saraiva, Moacir Franco de Oliveira, Antônio Chaves de Assis, Carlos Eduardo Bezerra de Moura

**Affiliations:** 1 Departamento de Ciências Animais, Universidade Federal Rural do Semi-Árido, Mossoró, RN, Brasil; 2 Departamento de Morfologia, Universidade Federal do Amazonas, Manaus, AM, Brasil; 3 Instituto Nacional de Pesquisas da Amazônia, Manaus, AM, Brasil; 4 Departamento de Morfologia, Universidade Federal do Rio Grande do Norte, Natal, RN, Brasil; 5 Faculdade de Medicina Veterinária e Zootecnia, Universidade de São Paulo, São Paulo, SP, Brasil

**Keywords:** aromatase, sex determination, turtle, reproduction

## Abstract

Sex steroid hormones are critical in gonadal differentiation in turtles. The gonads are not the only organs responsible for producing these hormones during this phase. Mesonephros play an important role in steroidogenesis. The present study aimed to investigate the presence of steroidogenic cells in mesonephros of *Podocnemis expansa* during gonadal differentiation and to evaluate their morphology and ultrastructure. Ten embryos of *P. expansa* were collected from 5 nests on day 36 of incubation, during spawning period on an artificial beach. Embryos were extracted from eggs by slicing the shell and euthanized. They were dissected under a stereoscopic microscope to collect the gonad-mesonephro complex, in which were fixed and subsequently processed for light microscopy, immunohistochemistry and transmission electron microscopy analysis. During histological analysis was observed mesonephros has typical morphological structure. Immunohistochemistry showed immunoreaction to aromatase in cells of intertubular space. Confirming these findings, it was possible to observe a type of intertubular cell in several regions of mesonephro, being more predominant in region close to blood vessels, distal and proximal tubules. In ultrastructural analysis these cells were characterized by having a clear, large, and rounded nucleus with evident nucleolus and cytoplasm rich in electron-dense droplets. This study demonstrated for the first time the presence of cells with morphological, immunohistochemical and ultrastructural characteristics similar to steroid-producing cells in P. expansa mesonephrons, suggesting that this organ may contribute to gonadal differentiation in this species.

## Introduction

*Podocnemis expansa,* known as the Amazonian turtle, is the largest freshwater turtle in South America (Vogt, 2008). The nesting of this species is influenced by the water levels of the river, in which spawning, and hatching are carried out in the dry season and the hatching of eggs coincides with the beginning of the rainy season and the rise of rivers (Vanzolini, 2003; Piña et al., 2006). This species lays an average of 92 eggs (63-134) and the hatching period is between 55 and 70 days (Bonach et al., 2011). It presents sexual determination influenced by incubation temperature (Temperature-dependent sex determination - TSD), in which high temperatures promote birth more females, while low temperatures increase male births (Valenzuela, 2001; Bonach et al., 2011). There is usually a time window known as thermosensitive period (TSP), during the second third of incubation period, in which physical temperature stimulus is transformed into biological stimulus acting on gonadal tissues and triggering sexual determination (Radhakrishnan et al., 2018).

During sexual determination, estrogen rate is a critical element for ovarian development in all vertebrate groups. In TSD species, this hormone is superior to temperature influences because estrogen-treated embryos generate females at temperatures appropriate to male development (Thomas et al., 1992).

Estrogen synthesis occurs in steroidogenic cells, in which the aromatase enzyme acts to convert the substrate from androgen to estrogen, playing a key role in biological functions that depend on this hormone, including sexual differentiation of developing vertebrate embryos (Matsumoto et al., 2014). There are reports that other extra-gonadal organs and tissues, such as mesonephros have been targets of temperature during gonadal differentiation and serve as sources of estrogen production (Barakat et al., 2016). However, so far, there are no studies that show participation of mesonephros in the production of estrogen in *P. expansa*. Thus, present study aimed to investigate presence of steroidogenic cells in mesonephro of *P. expansa* turtles at the beginning of gonadal differentiation, as well as to describe the morphology of these cells.

## Methods

In the present study, 10 embryos were evaluated at 36 days of incubation, onset of gonadal differentiation. These embryos were obtained from five *Podocnemis expansa* nests, during the spawning period on an artificial beach of the Aquatic Chelonian Research and Preservation Center (*CPPQA*). Collection was authorized under the Collection License of the Biodiversity Authorization and Information System (*SISBIO*/*ICMBio* 39472-4) and the Animal Research Ethics Committee of the National Amazon Research Institute (*CEUA-INPA* 025/2013).These samples are part of a larger project, previously developed, which investigated embryonic development and gonadal differentiation in *P. expansa,* in a natural environment with an incubation period of 58 to 64 days and an average incubation temperature of 30.3ºC. In this, the undifferentiated gonad was identified from the 14th day of incubation to the 34th day. From the 36th day of incubation, it was possible to identify the initial differentiation of the gonad into ovary and testis.

Embryos were collected and euthanized using 2.0 ml of intrapleuroperitoneally administered lidocaine hydrochloride. They were dissected under a stereoscopic microscope to collect the gonad-mesonephro (GM) complex, which were then fixed in 4% paraformaldehyde solution in phosphate buffer, for 12h.

After fixation, samples were submitted to histological processing according to Macêdo et al. (2018). The slides stained with Hematoxylin and Eosin. Images were then obtained by an Axioplan 2 light microscope coupled to an axcam MRc camera.

Histological sections were subjected to immunohistochemistry using the Rabbit anti-aromatase antibody – (AB18995) in a ratio of 1:250 and biotinylated secondary antibody to rabbit antibody (Goat Anti-Rabbit IgG H&L - AB205718) at a concentration of 1:500 as described by Macêdo et al. (2018). For negative controls, the polyclonal anti-Rabbit isotypic antibody (ab171870) was used. For antibody validation, sheep testis sections were used as a positive reaction control.

Samples were fixed in Karnovsky solution for 24h, then post-fixated with 1% osmium tethoxide for 2h. This was followed by dehydration in series of 15%, 30%, 50%, 70%, 95%, 100% (2x) graduated ethanol for 10 minutes and propylene oxide (2x) for 15 minutes each. Samples were incorporated at increasing concentrations of Durcupan-ACM Fluka^©^ resin at 4 °C for 4 days and polymerized at 60 °C for 72 h. Ultrathin 70 nm sections were prepared on a Reichert OM U3 Ultramicrotome, collected on 200 Mesh Formvar coated copper grids, then counterstained with 5% uranyl acetate followed by 0.5% lead citrate. They were then visualized under a 109 Zeiss EM Transmission electron microscope.

## Results

**Figure 1:**
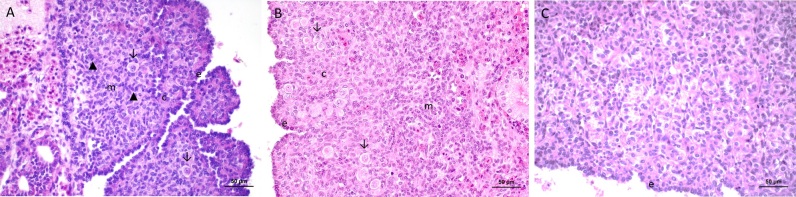
Photomicrograph of gonads at different stages of gonadal development. A - Undifferentiated gonad. B - Gonad in differentiation to ovary. C - Differentiating gonad to testis. Note the formation of tubular cords. e - epithelium. c - cortex. m - marrow. Arrow - primordial germ cell. Arrowhead - medullary cords.

The undifferentiated gonad had two distinct regions, the outer cortex in which primordial germ cells are preferentially located, and the inner medulla where the primitive sex cords are found. Cells were observed in the transition from the mesonephros to the medullary region of the gonad (Figure 1A).

From day 35 of incubation, it was possible to identify the beginning of ovarian and testis differentiation. The ovary had invaginations in its lining epithelium and composed of cylindrical cells. The two ovarian regions began to be defined and organized; the cortex was characterized by the presence of randomly distributed germ cells, and in the medullary region the sexual cords were still identified, however, a disorganization of the medulla was observed (Figure 1B).

In the testis differentiation, the parenchyma was characterized when the medullary cords were organized into tubular cords. Posteriorly, differentiation into seminiferous tubules is most evident by a thin basal lamina externally supporting the seminiferous tubule cells. At the end of differentiation, the testis had a thin epithelium, without invaginations, therefore with a regular appearance and lacking germ cells (Figure 1C).

**Figure 2:**
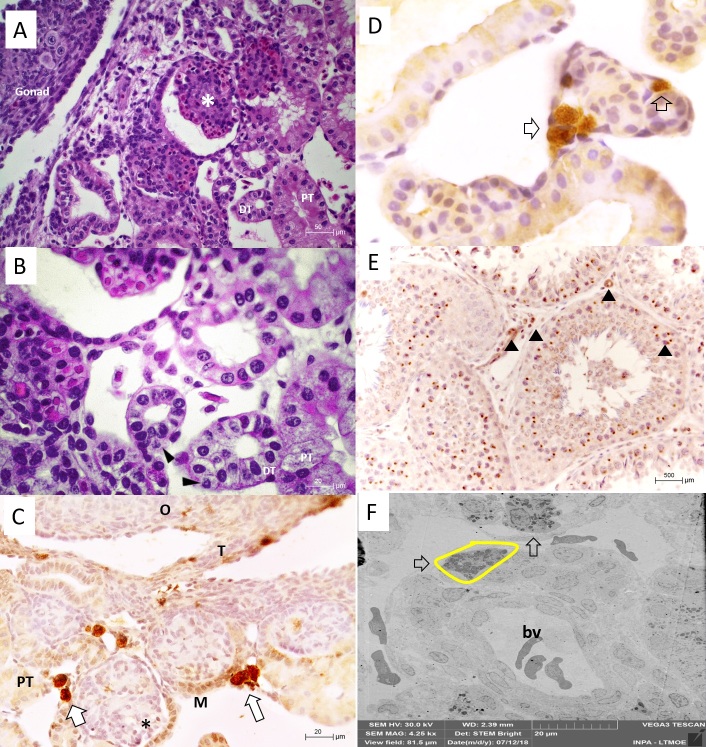
Gonad-mesonephron complex of P. expansa. A - General organization of the mesonephros. B – Steroidogenic cell (►) in the interstitial region of the mesonephron. C - Aromatase immunostaining in the mesonephros. Cells with strong aromatase immunoreaction (empty arrow). D - Higher magnification image of steroidogenic cells (empty arrows) near the renal tubules. E – Sheep testis sections as a positive immunoreaction control. Observe positive germ cells, Sertoli and Leydig cells (arrows head). F - TEM image. Steroidogenic cell (yellow outline) in the interstitial region between the proximal tubule and blood vessel (bv); Cytoplasm of steroidogenic cells rich in electron-dense lipid droplets (→). O - ovary; T - Transition region between mesonephros and gonads; M - Mesonephros. Glomeruli (*), Steroidogenic cell (►), Proximal (PT) and distal (DT) tubules.

At the beginning of gonadal differentiation, the mesonephros is characterized by the presence of renal corpuscles (RC) and renal tubules. The RC is composed of the glomerulus formed by a capillary network delimited by capsule. The epithelium of the proximal renal tubules (PT) was formed with tall cubic to cylindrical cells, while the distal tubule (DT) had short cubic cells (Figure 2A).

During this evaluation, was observed presence of round cells with nucleus occupying a large area with a low cytoplasm:nucleus ratio, dispersed throughout the entire length of the mesonephros (Figure 2B). These cells exhibited strong immunoreaction to aromatase (Figure 2C-2D).

In the ultrastructural analysis, it was possible to observe that this cell type was characterized by a clear, large, and rounded nucleus with an evident nucleolus, and cytoplasm rich in electrodense lipid droplets, located close to the blood vessels (Figure 2F).

## Discussion

The present work identified for the first-time cells with steroidogenic characteristics in *P. expansa* mesonephros during gonadal differentiation. It was a distinct cell type with rounded shape and exhibiting a low cytoplasm:nucleus ratio, compatible with the description of cells with steroidogenic activity (Campbell et al., 1996). These cells also showed immunoreactivity to aromatase, which is the main evidence of this activity.

Furthermore, these cells strongly stained for this enzyme were also found in the interstitial space, near blood vessels. This finding is common in cells with endocrine function, as the proximity to blood vessels favors the secretion of their products, possibly hormones (Schaeffer et al., 2011). It is known that high temperatures predispose the increase in aromatase enzymatic activity (Plewes and Burns, 2018), which would lead to the conversion of androgens into estrogens and favor the emergence of females in this species (Bonach et al., 2011).

In a complementary way, ultrastructural analysis revealed that the cells described as having a clear, large, rounded nucleus with an evident nucleolus and cytoplasm rich in lipids droplets, characteristics of high steroidogenic activity (Amsterdam and Rotmensch, 1987). These droplets are formed by cholesterol esters, used as precursors of sex steroid hormones (Plewes and Burns, 2018). In addition, these lipid structures have been reported in other steroidogenic cells such as leydig cells (Wang et al., 2015), large and small lutein cells (Plewes and Burns, 2018) and granulosa cells (Khor et al., 2014). In parallel, it was further observed that steroidogenic cells found in mesonephros have an ultrastructure similar to those have an ultrastructure similar to germline cells located in the gonads, (Soto-Suazo and Zorn, 2018), which also expresses aromatase (Miller and Bose, 2011).

## Conclusion

Cells with morphology indicative of steroidogenesis and strong immunoreaction to aromatase were observed in the mesonephro of *P. expansa*, at the beginning of gonadal differentiation. This finding indicates a possible role of this organ in this process.
